# The role of endoscopic surgery in the treatment of nasal inverted papilloma

**DOI:** 10.1016/S1808-8694(15)31124-1

**Published:** 2015-10-20

**Authors:** Guilherme de Toledo Leme Constantino, Tatiana T. Abdo, Fabrízio R. Romano, Richard L. Voegels, Ossamu Butugan

**Affiliations:** 1 MD. Otorhinolaryngologist. Endonasal endoscopic surgery fellow HC/FMUSP; 2 MD. Otorhinolaryngologist. Endonasal endoscopic surgery fellow HC/FMUSP; 3 PhD in Sciences - FMUSP, MD. Collaborator at the Department of Otorhinolaryngology HC/FMUSP; 4 Associate Professor of Otorhinolaryngology - FMUSP, Associate Professor of Otorhinolaryngology - HC/FMUSP; 5 Associate Professor of Otorhinolaryngology - FMUSP, Associate Professor of Otorhinolaryngology - HC/FMUSP

**Keywords:** endoscopic sinus surgery, inverted papilloma, recurrence

## Abstract

**Summary:**

The inverted papilloma is a benign neoplasm, prone to malignancy, and bearing a high rate of post-op recurrence. There is much debate in the literature concerning the issue that an endoscopic approach may offer a benefit over an external approach.

**Aim:**

Demonstrate the efficacy of an endonasal endoscopic approach in the treatment of inverted papilloma.

**Study design:**

Retrospective.

**Materials and Methods:**

Retrospective analysis of patients with inverted papilloma operated at the University Hospital - FMUSP from 1994 to 2004.

**Results:**

Twenty-eight patients' records were studied. Nine patients (32.1%) had tumor recurrence, one being operated via the endoscopic method and eight by external approach.

**Discussion:**

Krouse's staging system for inverted papillomas can facilitate both treatment planning and comparison of surgical outcomes. The use of the endoscopic approach resulted in fewer relapses than when the external one was used in cases with the same tumor staging.

**Conclusion:**

The use of the endoscope in this type of surgical treatment is an important success factor in the treatment of inverted papilloma.

## INTRODUCTION

The inverted papilloma is a benign neoplasia that has its origin in the lateral nasal wall epithelium. Histologically, it is characterized by an epithelial growth towards the stroma1. These are rare tumors, with an incidence of 0.5 to 1.5 cases per 100 thousand inhabitants^2^, corresponding from 0.5 to 4% of all nasosinusal tumors^3^.

They mainly affect male patients, between the 5th and 6th decades of life and cause nasal obstruction, rhinorrhea and epistaxis^4^. They have high rates of post-operative recurrence, which stresses the importance of an accurate tumor mapping and its complete exeresis^1^.

Thus, with the progress of endonasal endoscopic surgical technique, this treatment option for the nasosinusal inverted papilloma is under discussion, being compared to traditional external approaches. In the present study we compared the postoperative results of both surgical techniques, staging patients according to the classification proposed by Krouse^5^.

## MATERIALS AND METHODS

We studied the charts of all the patients diagnosed with nasosinusal inverted papillomas who were operated upon at the Department of Otorhinolaryngology of the University Hospital of the Medical School of the University of São Paulo (HC/FMUSP) between January, 1994 and April, 2004, after proper approval by the Hospital Ethics Committee. Twenty-eight charts had all the necessary information and minimum follow up of 4 months.

All the patients underwent preoperative CT Scan and were divided in four staging groups, based on the Krouse's5 classification for the nasosinusal inverted papilloma (Table 1, Table 2, Table 3, Table 4).

**Table 1:** Krouse's classification

T1	Tumor restricted to the nasal cavity
T2	Tumor restricted to the ethmoid sinus and medial/superior portion of the maxillary sinus
T3	Tumor involving the lateral or inferior portions of the maxillary sinus or frontal or sphenoid sinuses
T4	Tumor beyond nose and paranasal sinus boundaries or malignant disease

**Table 2:** Tumor staging

Krouse's Staging	% of patients
T1	07.1%
T2	25.0%
T3	64.3%
T4	03.6%

**Table 3:** Approach

Approach	% of cases
Endoscopic	42.85%
External	42.85%
Combined	14.30%

**Table 4:** % recurrence by staging

Staging	of recurrences/of cases	% Recurrence
T1	0/2	0%
T2	1/7	14,3%
T3	7/18	38,9%
T4	1/1	100%

The study includes information on patient's gender, tumor side, time from symptoms onset until medical consultation, nasal obstruction, rhinorrhea, epistaxis and headache, prior treatment, papilloma-associated squamous cell carcinoma areas, intra and post operative complications, recurrence and postoperative follow up.

Patients' treatment was always surgical, aiming at full tumor removal and also of the periosteum of the tumor insertion region. External, endonasal endoscopic, or a combined approach was decided upon based on tumor extension and site. Nonetheless, due to advances in endonasal endoscopic surgical techniques that have happened during the study period (1994-2004), there has been a trend towards external approach in the patients operated early on, and more endoscopic or combined approaches in the more recent cases, even in extensive tumors.

## RESULTS

Of the 28 studied patients with nasosinusal inverted papillomas, there was male prevalence - 21 cases (75%). Seven patients were females (25%). Tumor involved the right side in 13 patients (46.4%) and the left side in 15 (53.6%), and there was no bilateral involvement. Age varied from 18 to 70 years, with average of 52.9 years. Symptoms onset until consultation at the University Hospital - USP, varied much, between 3 and 240 months, with an average time span of 59.2 months. Regarding main symptoms, 27 patients presented nasal obstruction (96.4%), 20 rhinorrhea (71.4%), 13 epistaxis (46.4%) and 16 headache (57.1%). Six patients (21.4%) had already been operated upon in another service, one of them had undergone two operations and another one had had five surgeries.

Based on the Krouse's classification, considering preoperative paranasal sinus CT Scan and the presence of tumor malignancy, we had two patients in T1 (7.1%), seven cases in T2 (25.0%), 18 T3 (64.3%) and one T4 patient (3.6%) with squamous cell carcinoma in the pathology exam. External surgical approach was carried out via transmaxillary sublabial in 12 patients (42.85%). Twelve cases were operated upon only endoscopically (42.85%) and four of them underwent combined surgery - endoscopic and external with sublabial incision (14.3%). The two T1 cases were operated endoscopically (100%). Among the seven T2 patients: three suffered endoscopic surgery (42.85%), three external (42.85%) and one was combined (14.3%). Eighteen T3 cases: seven endoscopic (38.9%), eight external (44.4%) and three combined (16.7%). The only T4 case was operated through an external approach (100%).

The rate of surgical complications was low, there was one case of sphenopalatine artery injury (3.6%), without other intra-operative complications. Two patients (7.1%) had synechia in the postop, one in the middle meatus and another between the lower conchae and the nasal septum.

There was tumor recurrence in nine patients (32.1%), with diagnostic time between 4 and 45 months, 11 months in average. Average postop follow up was of 32.7 months, varying between 4 and 92 months. Among the nine cases of tumor recurrence, two had already been operated in another facility (22.2%). There was no recurrence in any of the two T1 cases. One among the seven T2 cases presented recurrence (14.3%), and this one was operated via the external approach. Seven patients among 18 T3 had recurrences (38.9%), with one endoscopic case (14.3%) and six external approaches (85.7%). The T4 patient had a recurrence (100%).

When we consider the number of recurrence cases divided by the total number of patients operated by surgical approach, separated by staging, we obtained: T2- no recurrence case among the three endoscopic cases and one among the three external approaches (33.3%). The only patient operated by the combined approach had no recurrence during the study period. T3- endoscopic: one recurrence among the seven cases (14.3%), external: six recurrences in eight operated cases (75%) and combined: no recurrence. T4- external: one case (100%). Globally speaking, the endoscopic approach presented recurrences in 12 operated cases (8.33%), the external approach had eight in twelve (66.66%) and the combined approach did not present recurrence (0%).

## DISCUSSION

Inverted papilloma is a benign tumor of the lateral nasal wall and the paranasal sinuses^1^,^6^, most prevalent in males2,4 - confirmed in our study with 75% of the cases being males. The major symptom described in the literature is unilateral nasal obstruction^4^,^7^ and our series had 96.4% of the patients with such complaint. The other more frequent symptoms in descending order of appearance are rhinorrhea, headache and epistaxis.

Although it is a benign lesion, the inverted papilloma is locally aggressive, bears high recurrence rates and is associated to squamous cell carcinomas in 5 to 15% of the cases^1^; for such reason, many surgeons adopt radical extra-nasal procedures as treatment of choice for these tumors^8^. However, with the progress in the endonasal endoscopic surgical technique, there has been much debate regarding possible benefits of such surgical approach over traditional ones such as lateral rhinotomy, degloving and sublabial approach^9^.

Krouse's classification for the inverted papilloma is based on preoperative CT Scan and the presence of malignant disease, allowing for surgical planning, but also a standardization to compare postoperative results^5^. Although CT Scan may overestimate the disease extension because it is not able to differentiate tumoral areas from inflammatory and secretion retention ones, 9, it still is a good assessment method. MRI has the advantage of better differentiating tumor from inflammatory tissue/secretion, however it is of higher cost and is also unable to differentiate the papilloma from a malignant tumor^10^.

The present review showed two cases staged as T1 (7.1%), and both of them were approached endoscopically, without recurrence and one T4 case (3.6%) with squamous cell carcinoma associated, operated externally and with postoperative tumor recurrence. Since treatment modality was the same in T1 and T4 cases, we are unable to compare surgical approaches.

Among the seven T2 cases, there was one recurrence among the three cases operated externally (33.3%) and there was no recurrence in the three endoscopic cases and in the combined approach patient. However, T3 group is the one with the largest number of patients and the one that allows better comparison among treatment modalities. Eighteen T3 cases were operated, seven via endoscopy, with one recurrence (14.3%); and eight externally with six recurrences (75%); and three cases operated via the combined approach, without recurrences, suggesting that the endoscopic approach may be beneficial in the nasosinusal papilloma surgery exclusively or in combination with the external access, since it allows better visualization of tumor extension and its insertion.

## CONCLUSION

Our ten year review with 28 cases of nasosinusal inverted papilloma pointed to a greater trend towards recurrence among the cases with more tumoral extension. The endonasal endoscopic approach used alone or in combination with the external access proved to be efficient and bearing lower tumor recurrence rates when compared to the external approach alone in cases classified as T2 and T3, according to Krouse's classification. Thus, we conclude that the endoscope use, during surgery, is an important tool in order to obtain success in the treatment of nasosinusal inverted papilloma.
